# A superconducting switch actuated by injection of high-energy electrons

**DOI:** 10.1038/s41467-021-21231-2

**Published:** 2021-02-24

**Authors:** M. F. Ritter, A. Fuhrer, D. Z. Haxell, S. Hart, P. Gumann, H. Riel, F. Nichele

**Affiliations:** 1 IBM Research Europe, Rüschlikon, Switzerland; 2 IBM T. J. Watson Research Center, Yorktown Heights, NY USA

**Keywords:** Superconducting properties and materials, Superconducting devices

## Abstract

Recent experiments with metallic nanowires devices seem to indicate that superconductivity can be controlled by the application of electric fields. In such experiments, critical currents are tuned and eventually suppressed by relatively small voltages applied to nearby gate electrodes, at odds with current understanding of electrostatic screening in metals. We investigate the impact of gate voltages on superconductivity in similar metal nanowires. Varying materials and device geometries, we study the physical mechanism behind the quench of superconductivity. We demonstrate that the transition from superconducting to resistive state can be understood in detail by tunneling of high-energy electrons from the gate contact to the nanowire, resulting in quasiparticle generation and, at sufficiently large currents, heating. Onset of critical current suppression occurs below gate currents of 100fA, which are challenging to detect in typical experiments.

## Abstract (web summary)

A recent finding of tuning critical current in metallic nanowires by application of small gate voltages seems at odds with general understanding. Here, Ritter et al. study similar nanowires and link the origin of the critical current suppression to tunneling of few high-energy electrons between gate and nanowire, ruling out direct tuning by electric fields.

## Introduction

Superconducting circuits, thanks to their ultra-low power consumption and high speed, offer great promise as building blocks for quantum computing architectures and related cryogenic control electronics. In this context, it is especially intriguing to develop switching devices that can be electrically tuned between a superconducting and a resistive state at high frequency. Ultimately, such a three-terminal device would enable novel functionalities for which no semiconducting counterpart exists, such as cryogenic switches, ultra-sensitive detectors, amplifiers, circulators, multiplexers, and frequency tunable resonators^1–8^. Several electrically controlled superconducting switches based on the injection of out-of-equilibrium quasiparticles in Josephson junctions have been realized^9–12^. However, Josephson junctions typically come with limited source-drain critical currents and the requirement to operate in magnetic field-free environments. Consequently, architectures that do not rely on Josephson junctions are subjected to intense study. Such pioneering approaches are based on three or four terminal devices where electrical currents^13^, locally generated Oersted fields^14^ or heat^15–17^ drive a superconducting channel normal. Finally, recent experiments suggest that moderate electric fields might affect superconductivity in metallic nanowires^18,19^. Controlling superconductivity in metallic devices via gate voltages would be appealing, however, a satisfactory explanation for the observed phenomena was not provided, yet.

Here, we report an experimental investigation of metallic nanowires subjected to electric fields. Our findings rule out any variation of superconducting properties as a direct consequence of the applied electric field, as suggested in refs. ^18,19^. On the other hand, we highlight the importance of tunneling and field emission from the gate electrode. Detailed measurements indicate that relatively few electrons, injected at energies several orders of magnitude higher than the superconducting gap, trigger the generation of a large number of quasiparticles and weaken superconductivity. This is in stark contrast to previously demonstrated devices actuated by quasiparticle injection at low energy^10,15,17^, where gate currents comparable to the device critical current (a few μA) were needed for switching. For larger gate currents, injected electrons locally increase temperature and drive the nanowires normal. We characterize the effect of electron injection into nanowires in terms of their critical current and its dependence on gate voltage, temperature, and magnetic field. This basic characterization is performed with different substrates and superconductors. We then investigate how injected quasiparticles influence superconductivity along the length of the channel in a region free of electric fields. After presenting the experimental observations, we will elaborate on their physical origins.

## Results

### Basic characterization

**Fig. 1:**
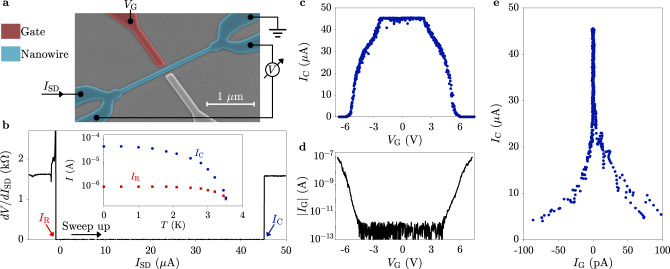
Operation of a metallic nanowire superconducting switch. **a** False-color scanning electron micrograph of a device identical to that under study, together with a schematic of the measurement setup. The silicon substrate is gray, the TiN nanowire blue and the gate electrode red. Another gate electrode (gray) was left grouded. **b** Differential resistance *dV*/*dI*_SD_ of the nanowire as a function of *I*_SD_, measured by sweeping up *I*_SD_ starting from −50 μA. Critical current *I*_C_ and retrapping current *I*_R_ are indicated. Inset: temperature dependence of *I*_C_ (blue dots) and *I*_R_ (red squares). **c** Critical current *I*_C_ in the nanowire as a function of gate voltage *V*_G_. **d** Absolute value of the gate current *I*_G_ flowing between gate and nanowire as a function of *V*_G_. A linear component *I*_G_ ~ 1 TΩ, attributed to leakage in the measurement setup, was subtracted from the data (see Supplementary Note 1). **e** Parametric plot of *I*_C_ vs. *I*_G_. Obtained from the data in **c** and **d**.

A typical device is shown in Fig. 1a together with its measurement setup: it consists of a 2 μm long, 80 nm wide TiN wire (blue) with a TiN side gate (red). Wire and side gate are separated by an 80 nm wide gap. Gates and nanowires were defined by electron beam lithography and dry etching of a TiN film deposited on an intrinsic Si substrate (gray). Measurements were performed by low-frequency lock-in techniques by passing a source-drain current *I*_SD_ in the nanowire and recording the resulting voltage *V*. Gate voltage *V*_G_ was applied via a source-measure unit, which also recorded the current *I*_G_ entering the gate contact. A second side gate (gray) was not operated in this work and left grounded. Further details on materials, samples fabrication, and measurement techniques are reported in the Methods Section. At *V*_G_ = 0, the nanowire showed a critical current *I*_C_ = 45 μA, measured sweeping *I*_SD_ in either direction starting in the superconducting state. In contrast, when sweeping *I*_SD_ from the normal state towards zero, superconductivity was re-established below the retrapping current *I*_R_ = 1 μA. Figure 1b shows the nanowire differential resistance *dV*/*dI*_SD_, measured while sweeping *I*_SD_ in the positive direction. The inset gives the temperature dependence of *I*_C_ and *I*_R_. The large difference between *I*_C_ and *I*_R_, especially marked at low temperature, is likely owing to self-heating when the nanowire is in the normal state, together with the difficulty in extracting heat via the substrate or the leads^20^. Figures 1c, d show *I*_C_ and *I*_G_, respectively, as a function of *V*_G_. For *V*_G_ ~ ±2.5 V, just before *I*_G_ reached detection level (~100 fA in our setup), *I*_C_ started decreasing. At *V*_G_ = ±5.5 V, where *I*_G_ ≈ ±1.5 nA, *I*_C_ vanished and the nanowire reached its normal state resistance *R*_N_ = 1.6 kΩ. The parametric plot of *I*_C_ vs. *I*_G_ shown in Fig. 1e indicates an extremely sharp suppression of *I*_C_ for small values of *I*_G_ (~50% of the suppression took place within the noise level for *I*_G_), followed by a slower decay which persisted up to *I*_G_ ~ 1.5 nA.

### Temperature and magnetic field dependence

**Fig. 2:**
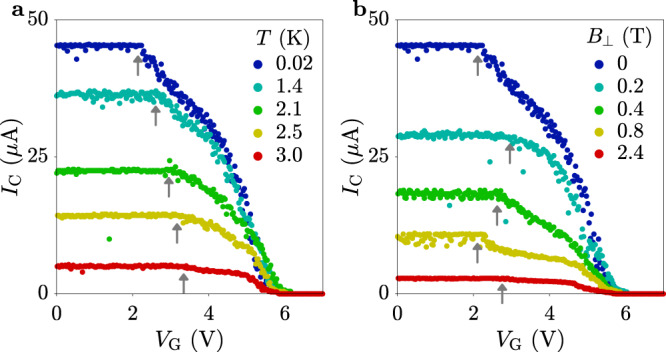
Temperature and magnetic field dependence. **a** Critical current *I*_C_ of the device presented in Fig. 1 as a function of gate voltage *V*_G_ for various temperatures *T* (see legend). **b** Critical current *I*_C_ of the device presented in Fig. 1 as a function of out-of-plane magnetic fields *B*_⊥_ (see legend). Gray arrows indicate the gate voltage where *I*_C_ starts decreasing.

Figure 2a, b show the gate voltage dependence of *I*_C_ for various temperatures *T* and out-of-plane magnetic fields *B*_⊥_, respectively. Neither temperature nor field affected the *I*_G_ vs. *V*_G_ characteristics of Fig. 1d (see Supplementary Note 1), and resulted in identical *V*_G_ values for complete suppression of superconductivity in the nanowires, up to the critical temperature and critical field. On the other hand, the increase of *I*_C_ suppression systematically moved to higher *V*_G_ for higher temperatures (see gray arrows). A more complicated dependence was observed as a function of *B*_⊥_.

### Critical current suppression in various superconductors

**Fig. 3:**
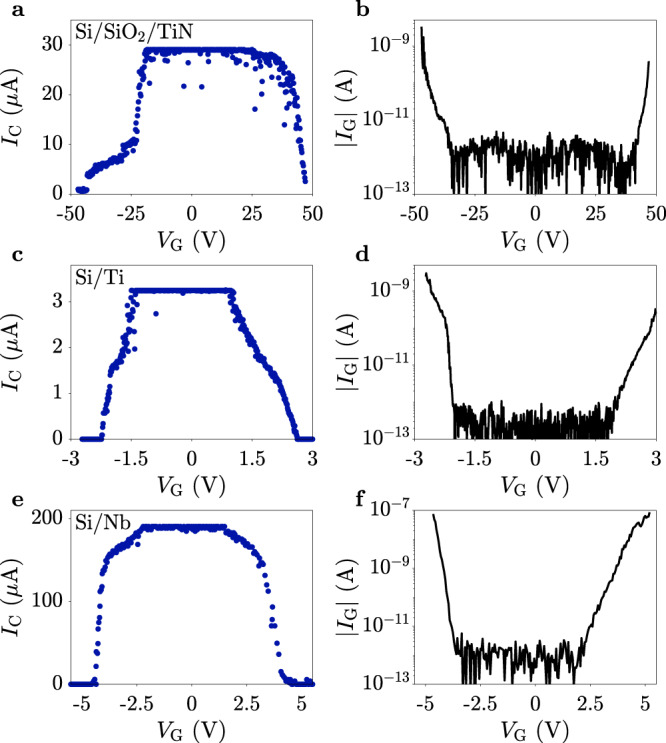
Critical current suppression in various superconductors. **a**, **b** Critical current *I*_C_ and gate current *I*_G_ as a function of gate voltage *V*_G_ for a TiN wire on a 25 nm thick SiO_2_ film thermally grown on Si substrate. The wire is 2 μm long, 80 nm wide, and 20 nm thick. **c**, **d** Critical current *I*_C_ and gate current *I*_G_ as a function of gate voltage *V*_G_ for a Ti wire on Si substrate. The wire is 2 μm long, 200 nm wide, and 30 nm thick. **e**, **f** Critical current *I*_C_ and gate current *I*_G_ as a function of gate voltage *V*_G_ for a Nb wire on Si substrate. The wire is 2 μm long, 200 nm, wide, and 13 nm thick.

Suppression of *I*_C_ concomitant to, or slightly anticipating, the onset of *I*_G_ above detection level was confirmed for over 20 TiN devices, characterized by various gate shapes, nanowire widths (40, 80, and 200 nm), nanowire lengths (650 nm, 1 and 2 μm), and gate-to-wire separations (80 and 160 nm, see Supplementary Note 3). Similar behavior was also observed on devices with a different substrate than Si or with a different superconductor than TiN. Figures 3a, b show measurements performed on a TiN device as that of Fig. 1a, but deposited on a 25 nm SiO_2_ layer thermally grown on Si. Despite the vastly different operational range of *V*_G_ with respect to that of Fig. 1, suppression of *I*_C_ still coincided with the onset of *I*_G_. Devices with a SiO_2_ interlayer further showed a characteristic asymmetry of the *I*_C_ vs. *V*_G_ curve, with a sharper suppression of *I*_C_ for negative than for positive *V*_G_. Given the sharp termination of the gate electrode, and the large electric field reached on SiO_2_ substrates, emission of electrons from the gate is expected to be easier for negative gate biases. In the present case, detection of small gate current asymmetries is hindered by spurious current leakage in the measurement setup for high gate biases. Figure 3c, d show *I*_C_ and *I*_G_, respectively, as a function of *V*_G_ for a Ti nanowire as that of Fig. 1a, but with 200 nm width and 30 nm thickness. In this case, the normal state was reached for *I*_G_ as low as 30 pA for positive *V*_G_. Figure 3e, f show *I*_C_ and *I*_G_, respectively, as a function of *V*_G_ for a Nb nanowire as that of Fig. 1a but with 13 nm thickness. Similarly to the previous cases, *I*_C_ started decreasing with *I*_G_ still below 100 fA. However complete suppression of *I*_C_ required *I*_G_ ≥ 20 nA. Overall, these results indicate that the switching mechanism presented here is generic, and not linked to specific superconductors or substrates. On the other hand, data also suggests that small gap superconductors (e.g., Ti) require considerably less gate current for switching to occur with respect to superconductors with larger gaps (e.g., TiN or Nb).

### Spatially resolved suppression of the critical current

**Fig. 4:**
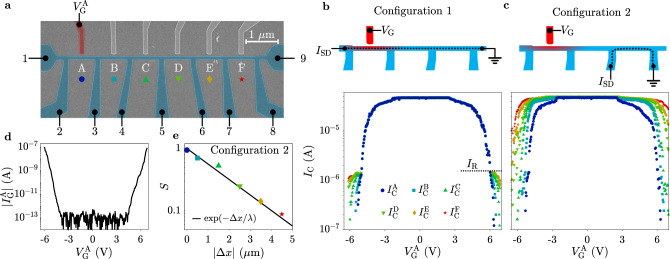
Spatially resolved suppression of the critical current. **a** False-color scanning electron micrograph of a device as that under study. Colors are as in Fig. 1a. **b** Top: schematics of measurement configuration 1, where *I*_SD_ always intersects the point of electron injection. Bottom: critical currents of the six segments as a function of gate voltage ${V}_{{\rm{G}}}^{{\rm{A}}}$. **c** Top: schematics of measurement configuration 2, where *I*_SD_ does not necessarily intersect the point of electron injection. Bottom: critical currents of the six segments as a function of gate voltage ${V}_{{\rm{G}}}^{{\rm{A}}}$. **d** Absolute value of gate current ${I}_{{\rm{G}}}^{{\rm{A}}}$ as a function of gate voltage ${V}_{{\rm{G}}}^{{\rm{A}}}$. **e** Suppression factor *S* as a function of the distance between gate and nanowire segment, calculated from the data in **c** for ${V}_{{\rm{G}}}^{{\rm{A}}}=6\ {\rm{V}}$. The solid line is a fit to an exponential, resulting in a characteristic length scale *λ* = 1.8 μm.

Measurements presented so far were conducted in relatively short nanowires, where sharp transitions from zero resistance to the normal state were observed. We complement these observations with measurements on a long, multi-terminal nanowire, which allow us to investigate how superconductivity is affected away from the electron injection point, along the nanowire length. The device shown in Fig. 4a consists of six TiN segments of 1 μm length and 80 nm width (named *A* to *F*). Each segment *j* is controlled by a nearby gate, with gate voltage ${V}_{{\rm{G}}}^{{\rm{j}}}$ and corresponding gate current ${I}_{{\rm{G}}}^{{\rm{j}}}$. In a first measurement configuration (Configuration 1), schematically shown in Fig. 4b, *I*_SD_ was passed between contacts 1 and 9, that is the DC current is the same for every segment. As *I*_SD_ was ramped, voltages *V*_*j*_ across the six segments were simultaneously recorded. Critical currents ${I}_{{\rm{C}}}^{j}$, defined as the values of *I*_SD_ where segment *j* turned resistive, are reported in Fig. 4b as a function of ${V}_{{\rm{G}}}^{{\rm{A}}}$, with the corresponding gate current ${I}_{{\rm{G}}}^{{\rm{j}}}$ shown in Fig. 4d. Configuration 1 highlights two regimes. For *I*_SD_ > *I*_R_, switching in all the segments happened simultaneously. For *I*_SD_ < *I*_R_, switching was sequential: the further away a segment was from the biased gate, the larger was the gate current required to suppress its critical current. We contrast this behavior with the results obtained using Configuration 2, schematically shown in Fig. 4c. In this case, *I*_SD_ is routed in one segment only. The critical currents of the six segments were extracted in six separate measurements as ${V}_{{\rm{G}}}^{{\rm{A}}}$ was biased (see Fig. 4c). Routing *I*_SD_ far from the electron injection point avoids the simultaneous switching observed in Fig. 4b, highlighting instead spatial dependence of the critical current also for *I*_SD_ > *I*_R_. In Fig. 4e, we plot the critical current suppression factor *S* as a function of distance Δ*x* between injection point and segment. The suppression factor for a segment *j* is defined as ${S}^{j}=({{I}_{0}}^{j}-{{I}_{{\rm{C}}}}^{j})/{{I}_{0}}^{j}$, where ${{I}_{0}}^{j}$ is the critical current of a segment for zero gate voltage. A fit to an exponentially decaying function $\exp (-{{\Delta }}x/\lambda )$ (solid line in Fig. 4f), yields a characteristic decay length *λ* ~ 1.8 μm.

## Discussion

After presenting the experimental results, we now discuss the origin of the observed phenomena. Injected electrons reach the superconductor in a deeply out-of-equilibrium state, with energies of the order *eV*_G_, much larger than the superconducting gap Δ (Δ = 500 μeV for TiN^21^). As each electron relaxes to the gap edge by inelastic scattering with other electrons and phonons, up to *eV*_G_/Δ ~ 10^5^ quasiparticles are generated within the nanowire. A sufficiently high concentration of quasiparticles drives the nanowire normal by quenching the superconducting gap^22^ and suppression of the depairing critical current^23^, leading to a switch to the normal state. The closer *I*_SD_ is to *I*_C_, the more sensitive the device becomes, so that relatively few injected electrons can trigger a normal state transition. Indeed, half of the suppression of *I*_C_ takes place for gate currents below the noise floor of our setup, where the power provided by the gate voltage source is less than 300 fW and unlikely to result in any relevant temperature increase. Indeed, a temperature increase ~1.5 K would be needed for an appreciable variation of *I*_C_ to be detected (see Fig. 1b). This can be is excluded at such small gate currents. Such behavior is reminiscent of superconducting nanowire single-photon detectors (SNSPDs)^24,25^, where the strike of a visible or infrared photon promotes a single electron to high energy, which in turn triggers the generation of a large amount of quasiparticles as it relaxes. In the present case, high-energy charge carriers are provided directly by the gate current. Owing to their close proximity, gate, and nanowire are coupled by phonons, so that dissipation of the injected energy and generation of quasiparticles occurs on both sides. Figures 2a, b show rich physics at low values of *V*_G_, with the initial suppression of *I*_C_ moving to higher and higher gate voltages as temperature increases (see gray arrows). This behavior presumably reflects the increase of quasiparticle density in the wire with temperature, requiring more electrons to be injected before a sizable effect on *I*_C_ is observed. Systematic studies of the more complicated variations of *I*_C_ vs. *V*_G_ as a function of *B*_⊥_ could shed new light on the physics of field repulsion and vortex penetration in nanowires^26^.

For gate currents several orders of magnitude larger, the power provided to the device is significant and likely to result in an increase of the local lattice temperature. We estimate the minimum power required for keeping the nanowire in the normal state as ${P}_{{\rm{R}}}={{I}_{{\rm{R}}}}^{2}{R}_{{\rm{N}}}$, which is Joule heating in the normal state and at the retrapping current. For the device of Fig. 1 we obtain *P*_R_ = 1.6 nW. This power is similar to one provided by the gate voltage source at the point where superconductivity is suppressed *P*_G_ = *V*_G_*I*_G_. For the device of Fig. 1, we obtain *P*_G_ = 6.1 nW. The difference between *P*_R_ and *P*_G_ is readily accounted for by considering that a significant fraction of *P*_G_ is not dissipated in the nanowire but in the gate electrode and in the surrounding environment. Furthermore, quasiparticles generated within the nanowire spread over a distance longer than the nanowire length, so that a fraction of them thermalizes in the leads (see the following discussion). The relation *P*_G_ ≈ 4*P*_R_ is closely followed also for the devices of Fig. 3. The consistency is remarkable considering that dissipated power in the Nb wire (*P*_R_ = 29 nW) is three orders of magnitude larger than for the Ti wire (*P*_R_ = 23 pW).

After determining that small currents of high-energy electrons are responsible for weakening the superconducting properties, we discuss in more detail how the transition to the normal state takes place. The device of Fig. 1 showed a sharp transition from superconducting to its normal state resistance for any gate voltage. This behavior might appear surprising considering that the gate acts on a short portion of the nanowire. With reference to Fig. 4, we demonstrated that the sharp transition to the normal state resistance is a result of the measurement configuration. Indeed, *I*_C_ is first reduced in a region close to the point of electron injection. As *I*_SD_ is increased, that region switches to the resistive state and becomes a hotspot due to the large *I*_SD_ flowing in the nanowire. For *I*_SD_ > *I*_R_ the hotspot warms up the surrounding metal via Joule heating, resulting in a further spreading of the normal region. This process rapidly turns normal the entire nanowire length along the path of *I*_SD_. For *I*_SD_ < *I*_R_ the power dissipated in the hotspot is insufficient to trigger the transition to the normal state in the nearby metal. In this case, considerable gate currents are needed for influencing the regions of the nanowire, which are furthest away via diffusion of energetic quasiparticles and heat. In Configuration 2, *I*_SD_ does not intersect the point of electron injection (except for segment A) and simultaneous switching is prevented and the critical current is lowest at the point of injection and restored at large distances. The characteristic length scale of 1.8 μm is presumably related to the diffusion length of long-lived quasiparticles. A framework for calculating quasiparticle density profiles has been put forward for SNSPDs in ref. ^26^ and is compatible with our experimental results.

Recent work argued on the effect of electric fields on the critical current of metallic nanowires, using a similar device as that of Fig. 1a^18,19^. From a qualitative standpoint, the modulation of *I*_C_ we observe strongly resembles data in refs. ^18,19^, including ambipolar behavior, response to temperature and magnetic field, and spacial suppression of the supercurrent reduction. We note that electric field modulation of superconductivity has been previously demonstrated in metallic thin films^27–29^, but changes of critical temperature by less than one percent required significantly stronger electric fields than those applied here. Investigating multiple material combinations and comparing local and non-local measurement configurations allow us to readily exclude any electric field-induced suppression of superconductivity in our devices. First, gate voltages are too small to generate a sizable electric field (see Supplementary Note 3 for a different gate design leading to identical results). Second, suppression of superconductivity is always correlated to the onset of gate currents, irrespective of the applied gate voltage. Third, non-local responses extend far beyond the gate induced electric field, to a distance where only quasiparticle diffusion and phonons are relevant (see Fig. 4). We further note that the gate current needed for affecting superconductivity is especially low for small gap superconductors on highly insulating substrates, the platform used in refs. ^18,19^. For large gap superconductors on semi-insulating substrates, i.e., the main focus of this work, the lower energy of emitted electrons, together with the requirement for higher quasiparticle density to suppress superconductivity, make required gate currents larger and their detection more feasible.

Injection of high-energy electrons in superconducting elements might be employed for the realization of fast and electrically controlled superconducting switches. As devices properly function also in the limit of *I*_SD_ = 0, self-resetting from normal to superconducting state is not inhibited by self-heating^17^, a limitation of other superconducting devices. The quasiparticle relaxation length limits the extent of the segment that can be switched, and consequently the largest normal state resistance of the device. Alternatively, nanowires of arbitrary length can be operated by choosing *I*_SD_ > *I*_R_^13,15^. We expect the switching time to be fast, presumably limited by quasiparticle recombination (<100 ps)^15^. In our case, we measured a switching time below 100 ns, limited by the setup in use (see Supplementary Note 4).

In conclusion, we demonstrated quenching of superconductivity in metallic nanowires via gate currents several orders of magnitude smaller than the source-drain critical current and described the physical mechanisms responsible for this behavior. Devices studied here could serve as tools for novel studies of quasiparticle physics at unprecedented energy scales and in the limit of no current flowing in the nanowire. Furthermore, combining length dependence studies as in Fig. 4 with time-resolved measurements, will provide a novel tool to investigate quasiparticle dynamics and thermal effects.

## Methods

### Sample fabrication

Devices were obtained on either intrinsic Si substrates or intrinsic Si substrates with a 25 nm thermally grown SiO_2_ top layer. Both high and low resistivity Si resulted in similar device performance at low temperature in terms of gate currents vs. gate voltages. Just prior to the deposition of the superconductor, Si chips were immersed in a buffered HF solution for removing the native Si oxide. Nanowires were defined by electron beam lithography, as detailed below. After the nanowires were fabricated, Ti/Au bonding pads, placed ~170 μm away from the active region of the devices, were defined by optical lithography, thermal evaporation, and lift-off.

TiN wires were obtained from a 20 nm TiN film was deposited by DC reactive magnetron sputtering. A 50 nm thick layer of hydrogen silsesquioxane (HSQ) based negative tone resist was used as mask. After resist development, unprotected TiN areas were removed by inductively coupled plasma etching in HBr plasma. After etching of the TiN, HSQ was removed by immersion in a diluted hydrofluoric acid solution. Characterization of the TiN film gave a resistivity of 68Ω per square, a critical temperature of 3.7 K and a critical out-of-plane magnetic field of 3.5 T. These properties remained unchanged in the completed devices.

Ti wires were defined by electron beam lithography on a positive tone polymethyl methacrylate mask, electron beam evaporation of a 30 nm thick Ti film and lift-off. Ti evaporation was performed at a base pressure of 10^−9^ mbar and at a deposition rate of 1 nm s^−1^. The high deposition rate was chosen to minimize contamination of the Ti film during evaporation^30^. The Ti wire of Fig. 3c had a critical temperature of 220 mK, a normal state resistance of 74Ω and a retrapping current of 560 nA.

Nb wires were obtained by sputtering of a 13.5 nm film on intrinsic Si substrates and following a fabrication similar to that described above for TiN. Dry etching was performed with Ar/Cl_2_ plasma. The Nb wire of Fig. 3e had a normal state resistance of 655Ω and a retrapping current of 7.6 μA.

### Electrical measurements

Unless differently specified, measurements were performed in a dilution refrigerator at the base temperature of 10 mK. Low-pass RC filters and microwave pi-filters were installed along each line. A DC source-drain current *I*_SD_, superimposed to a small AC component of 30 nA and 113 Hz was applied to the nanowire via large bias resistors. The AC differential voltage *V* across the nanowire was then recorded with lock-in amplifiers with 10 MΩ input impedance and used to calculate the differential resistance *dV*/*dI*_SD_. Measurements were recorded with *I*_SD_ as the fast axis, sweeping from zero to positive values. This allowed initializing the wires to the superconducting state before each sweep started. Gate voltages were applied with a Keysight B2902A source-measure unit, which also recorded the current entering the gate contact. To avoid damaging the devices, a compliance of ±95 nA was chosen. A linear contribution of ~1 pAV^−1^, associated with spurious leakage paths in our setup, was subtracted from the *I*_G_ measurements shown in the Main Text (see Supplementary Note 1). To avoid potential contributions from displacement currents or hysteresis, *I*_G_ values were recorded by sweeping *V*_G_ from 0 V towards either positive or negative voltages and waiting times in excess of 30 s were allowed. Plots as that of Fig. 1c, d were then obtained by merging two data sets at *V*_G_ = 0 V. In case a second gate was present and left grounded, as for the device in Fig. 1a, it was verified that most of the gate current was flowing from the energized gate to the nanowire and not to the grounded gate.

In a standard measurement configuration, *I*_SD_ is sourced on one side of the sample by applying a voltage to a large resistor, whereas the other side of the sample is connected to ground. This asymmetric configuration results in lifting of the nanowire potential by *I*_SD_*R*_L_, where *R*_L_ is the resistance of the line connecting the nanowire to ground (2.2 kΩ in our case). This effect is typically negligible, except for samples where a large *I*_SD_ is required (see Fig. 3e). A first approach is to use a symmetric current biasing configuration, as shown in Supplementary Fig. 4. However, using large resistors on both ends of the nanowire strongly reduces the available bandwidth. Alternatively, this effect can be accounted for by representing critical currents on the virtual voltage axis ${{V}_{{\rm{G}}}}^{* }={V}_{{\rm{G}}}-{I}_{{\rm{SD}}}{R}_{{\rm{L}}}$.

## Supplementary information

Supplementary Information

## Data Availability

The data that support the findings of this study are available from the corresponding authors on reasonable request.
