# Supplementary material for: A superconducting switch actuated by injection of high-energy electrons
Source: Nat Commun. 2021 Feb 24;12:1266. doi: 10.1038/s41467-021-21231-2 (PMC7904938; doi:10.1038/s41467-021-21231-2)
Supplement: Supplementary file 1 — Supplementary Information [file 41467_2021_21231_MOESM1_ESM.pdf]

Supplementary Information: A superconducting switch actuated by injection of high energy electrons

M. F. Ritter,[1] A. Fuhrer,[1,*] D. Z. Haxell,[1] S. Hart,[2] P. Gumann,[2] H. Riel,[1] and F. Nichele[1,†]

[1] *IBM Research Europe, Säumerstrasse 4, 8803 Rüschlikon, Switzerland*
[2] *IBM T. J. Watson Research Center, Yorktown Heights, NY, USA*

(Dated: December 19, 2020)

SUPPLEMENTARY NOTE 1: PROCESSING OF THE GATE CURRENT

Measurements of the gate current I_G, flowing between gate and nanowire channel were obtained with a Keysight B2902A source-measure unit and processed by the numerical technique described below. The source-measure unit applied a voltage V_G to the gate contact and read the current I_G flowing into the setup. An example of an I_G vs. V_G curve measured with this technique, referred to as Method 1, is shown in Fig. 1 (green dots). When measuring with Method 1, we describe the DC current flowing into the electrical setup as sum of two components: the current that actually flows into the gate electrode and reaches the nanowire, and the current that is lost before reaching the gate by spurious leakage paths present in the cryostat. The first current component is expected to depend exponentially on V_G, the second was found to be approximately linear. That is, for small gate voltages, the current flowing to spurious paths and not reaching the gate voltage is dominating. The following numerical procedure was applied to extract the current contribution that actually reaches the nanowire. First, a current offset related to the measurement device is subtracted from the data (typically within ±5 pA), so that $I_G = 0$ for $V_G = 0$. Second, a line is fit to the experimental $I_G(V_G)$ curve for small values of V_G (dotted black line in Fig. 1). Third, the obtained line is subtracted from the experimental curve in the entire V_G range, resulting in the red triangles in Fig. 1. This procedure typically results in gate currents which are within the noise level of our setup at low V_G, and then increase exponentially at large V_G. The linear component that we subtracted corresponds to a resistance of about 1 TΩ. Testing different parts of our setup individually showed that this spurious resistance is predominantly associated with the low frequency twisted pair wires that bring the signal from room temperature to the mixing chamber stage, and is present also when no device is connected. In our experience, such a high value of resistance to ground is indicative of a good DC measurement setup.

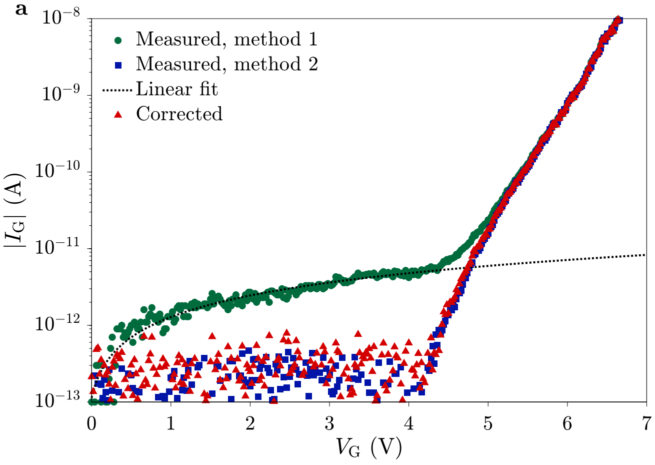

Supplementary Figure 1. **Processing of the gate current.** Measurement and processing techniques for the gate current I_G as a function of gate voltage V_G. Green dots are measured by Method 1 (see text), blue squares by Method 2 (see text). The dotted black line is a linear fit to the green dots for $Vg \leq 3.5$ V. Red triangles are obtained by subtracting the dotted line from the green dots.

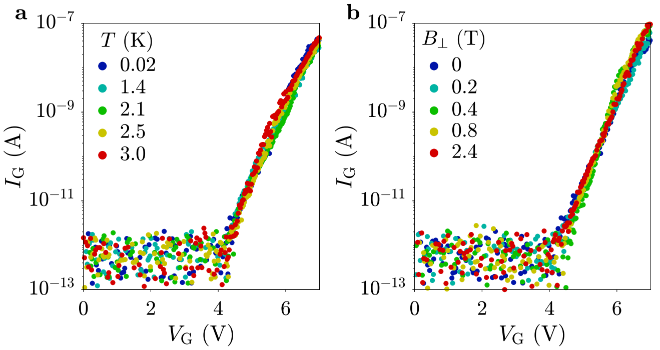

Supplementary Figure 2. **Temperature and magnetic field dependence of the gate current. a** Gate current I_C as a function of gate voltage V_G simultaneously measured as in Fig. 2(a) of the Main Text. **B** Gate current I_C as a function of gate voltage V_G simultaneously measured as in Fig. 2(b) of the Main Text.

To verify the validity of this numerical procedure, we measured the current with a second technique, referred to as Method 2. In Method 2, all contacts to the nanowire channel are left floating except for one, which is grounded via a low impedance IV converter (Basel Physics SP 983, with feedback resistance set to 1 GΩ). The gate current I_G injected into the nanowire channel flows into the IV converter and gives rise to voltage output of $I_G \times 1$ GΩ. The current I_G simultaneously measured with Method 2 is also shown in Fig. 1 (blue squares) and is essentially

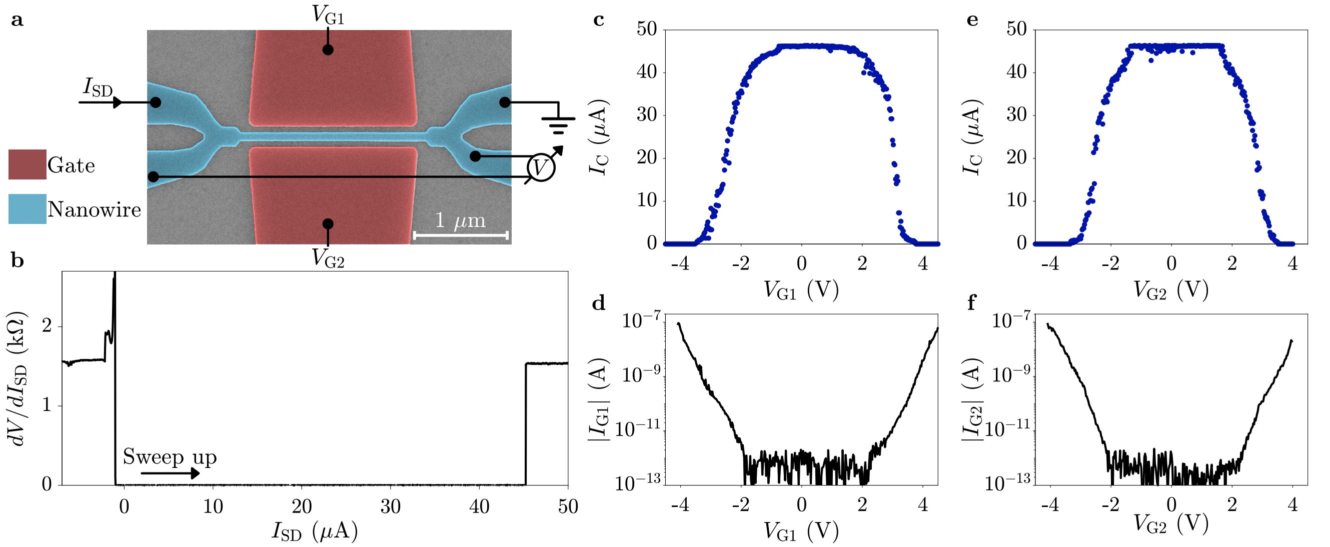

Supplementary Figure 3. **Measurements of a device with wide side gates. a** False-color scanning electron micrograph of the device under study and schematics of the measurement setup. The nanowire has been colored blue and the side gates red. **b** Nanowire resistance dV/dI_{SD} as a function of source-drain bias current I_{SD}. The measurement highlights the critical current $I_{\mathrm{C}} = 47\ \mu$A and the retrapping current $I_{\mathrm{R}} = 1\ \mu$A. **c** Critical current I_{C} as a function of gate voltage V_{G1}. **d** Gate current I_{G1} as a function of gate voltage V_{G1}. **e** Critical current I_{C} as a function of gate voltage V_{G2}. **f** Gate current I_{G2} as a function of gate voltage V_{G2}.

identical to that processed with the numerical technique described above. Adopting Method 2 throughout this work would however not be possible, as the large source-drain currents needed to reach the critical current would result in overloading of the IV converter. For example, the current to voltage gain of 1 GΩ allows for a maximum input current of 10 nA, while typical source-drain critical currents extend up to 200 μA. Operating with lower current-to-voltage conversion gain, such as 100 kΩ, would not provide enough resolution to detect small gate currents.

SUPPLEMENTARY NOTE 2: TEMPERATURE AND FIELD DEPENDENCE OF THE GATE CURRENT

Figure 2 of the Main Text shows the critical current I_{C} as a function of gate voltage V_{G} for the device of Fig. 1 of the Main Text measured at various temperatures and fields. In Fig. 2(a) and (b) we show the simultaneously measured gate currents I_{G}. Data indicate that I_{G} is unaffected by both temperatures and magnetic fields.

SUPPLEMENTARY NOTE 3: MEASUREMENT OF A DEVICE WITH LARGE GATES

Data shown in the Main Text was obtained on nanowires where gates were relatively narrow (of the order of 100 nm or less) and terminated with a sharp tip. In Fig. 3 we present measurements obtained on a nanowire as that of Fig. 1(a) of the Main Text, but with 2 μm wide gates. A false-colored scanning electron micrograph of the device is shown in Fig. 3(a), together with the measurement setup. The nanowire is colored in blue and the two gates in red. The gates are separately operated with gate voltages V_{G1} and V_{G2}, respectively. The response to a source-drain current was characterized in Fig. 3(b) by sweeping I_{SD} up from the resistive state. As for the wire in Fig. 1(a) of the Main Text, the critical current was $I_{\mathrm{C}} = 47\ \mu$A and the retrapping current $I_{\mathrm{R}} = 1\ \mu$A. The source-drain critical current I_{C} as a function of gate voltage V_{G1} is shown in Fig. 3(c), with the corresponding gate current I_{G1} shown in Fig. 3(d). Equivalent measurements performed as a function of gate voltage V_{G2} are shown in Figs. 3(e) and (f). For both V_{G1} and V_{G2}, full suppression of I_{C} was reached at about ± 4 V, corresponding to gate currents of about ± 1 nA.

SUPPLEMENTARY NOTE 4: TIME-RESOLVED MEASUREMENTS

Here we present time-dependent measurements, indicating fast switching operation as a function of V_{G} and the ability of the device to self-reset from normal to superconducting state. A schematic of the electrical setup used for those measurements is shown in Fig. 4(a), which allows for the simultaneous application of low frequency and high frequency signals (colored blue and red, respectively). The device was mounted on a sample holder with

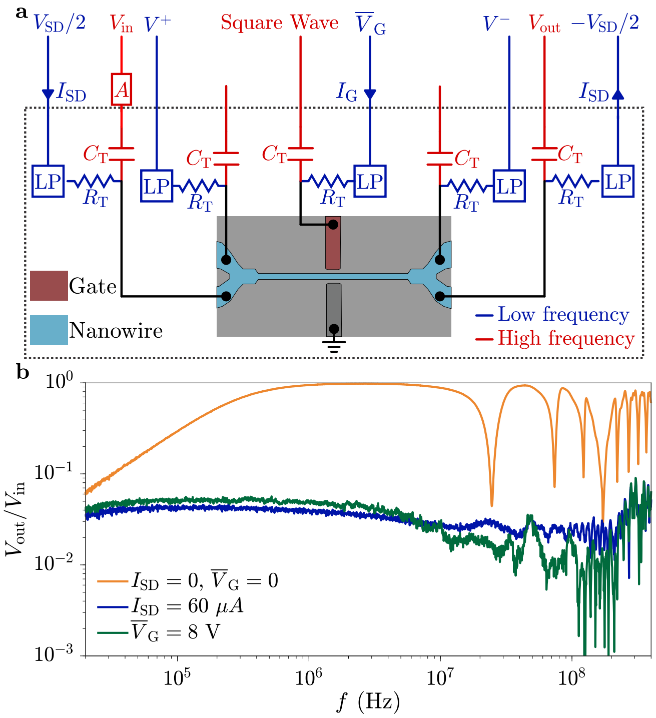

Supplementary Figure 4. **High frequency measurements.** **a** Schematic measurement setup for the high frequency experiment presented in Fig. 5 of the Main Text. The dashed box divides the 10 mK (inside) and room temperature (outside) apparatus. Lines for low and high frequency signals are indicated in blue and red, respectively. Low temperature bias-Ts with components R_T and C_T were connected to the four leads of the wire and a side gate. Low frequency cables were filtered by low-pass (LP) filters. Symmetric application of low frequency voltage $\pm V_{SD}/2$ results in the flow of a current I_{SD}. Fast signals V_{in} and V_{out} are applied and recorded, respectively, thorough 50 Ω ports. **b** Frequency dependent V_{out}/V_{in} signal measured with the nanowire in the superconducting state (orange), in the normal state as a consequence of a large source-drain current (blue), and in the normal state as a consequence of a large gate voltage (green).

resistive bias-Ts (resistance $R_T = 50$ kΩ and capacitance $C_T = 22$ nF). The low frequency lines (resistive twisted pairs) passed through RC low pass filters on the sample holder (LP in Fig. 3(a)) and additional RC filters and high frequency pi-filters at the mixing chamber level (not shown), resulting in an additional line resistance $R_L = 2.5$ kΩ. A voltage bias V_{SD}, symmetrically applied between two low frequency inputs, resulted in a source-drain current of approximately $I_{SD} = V_{SD}/(2R_T + 2R_L)$. Application of a symmetric bias ensured the nanowire potential was constant with respect to the gate potential as I_{SD} varied. Low frequency voltage signals V^+ and V^- were used to calculate the nanowire four terminal resistance as $R = (V^+ - V^-)/I_{SD}$.

The device transmission was measured via a lock-in amplifier (Zurich Instruments UHFLI, with input and

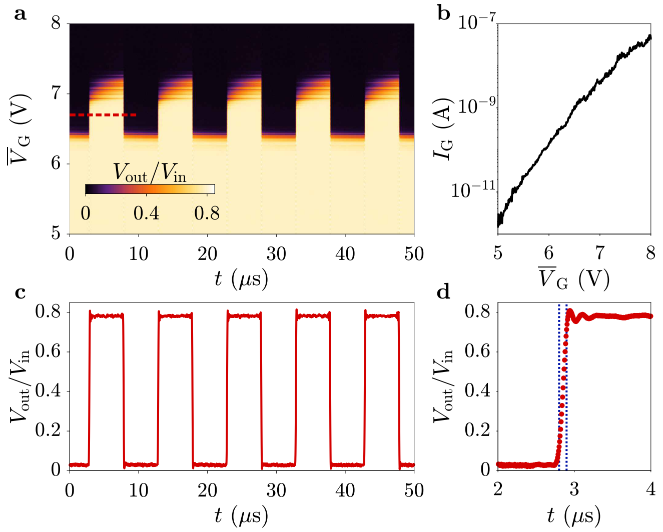

Supplementary Figure 5. **Fast switching in a metallic nanowire.** **a** Time-dependent switching characteristics of a device as that of Fig. 1(a) of the Main Text as a function of DC gate voltage $\overline{V}_G$. A 100 kHz square wave with peak-to-peak amplitude of 1 V was added to $\overline{V}_G$. **b** Gate current I_G as a function of $\overline{V}_G$ measured simultaneously to the data in (a). **c** Line-cut of the data in (a) for $\overline{V}_G = 6.7$ V (see red line). **d** Zoom-in of the data in (c) in proximity to a normal to superconducting state transition. Dashed vertical lines indicate the 10% to 90% amplitude transition, corresponding to 90 ns. This value is limited by the measurement bandwidth of the setup in use and serves as upper limit for the device switching time.

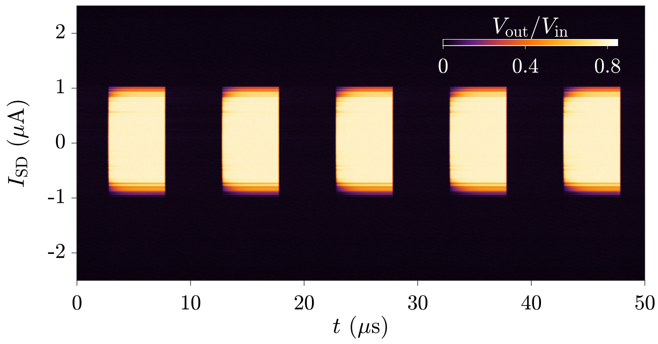

Supplementary Figure 6. **Demonstration of self-resetting.** Switching operation as a function of the DC source-drain current I_{SD}. Self-resetting was possible for I_{SD} values smaller than the nanowire retrapping current.

output set to 50 Ω impedance.) by applying a voltage V_{in} through a −80 dB attenuator (A) and recording the resulting voltage V_{out}. The ratio V_{out}/V_{in} is shown in Fig. 4(b) as a function of frequency f for three situations. In orange is the situation where the wire was superconducting, meaning $I_{SD} = 0$ and $V_G = 0$. In blue is the situation in which the wire was turned normal by means of a DC current $I_{SD} = 60 \mu$A, larger than the nanowire critical current $I_C = 50$ μA. In green is the situation in

which the nanowire was turned normal by the application of a DC gate voltage $\overline{V}_{\mathrm{G}} = 8$V. As expected, for sufficiently high frequency the device transmission in the superconducting state approaches unity. Deviations however occur at specific frequencies, presumably due to the fact that the device was not designed to operate at high frequencies. Measurements shown below were performed at a frequency of 250 MHz.

Using the low temperature bias-Ts, a square wave signal was superimposed to the low frequency gate voltage $\overline{V}_{\mathrm{G}}$. The ratio between the transmitted voltage V_{out} and the voltage input to the measurement setup V_{in} is shown in Fig. 5(a) as a function of time t and $\overline{V}_{\mathrm{G}}$, with the time-averaged gate current shown in Fig. 5(b). Clear switching operation was achieved within a 500 mV interval around $\overline{V}_{\mathrm{G}} = 6.7$ V, corresponding to a gate current of 1 nA. Figure 5(c) shows a line cut of Fig. 5(a) for $\overline{V}_{\mathrm{G}} = 6.7$ V, demonstrating fast and reproducible switching between two impedance states. A zoom-in close to a rise point is shown in Fig. 5(d), with dashed lines marking the transition between 10% and 90% of the step height, which takes place in 90 ns (similar results are obtained for the decay time). Such transient equals three times the time constant of the lock-in amplifier used for these measurements (30 ns) and is taken as the shortest switching time measurable with the setup in use, and as the upper limit for the device response time. Future work will take advantage of samples specifically designed for microwave measurements [1] and correlation techniques [2] to test the ultimate switching speed of the device.

Our superconducting switch has the remarkable property to operate without the need of a DC current I_{SD} flowing in it. Measurements shown in Fig. 5 were obtained with $I_{\mathrm{SD}} = 0$, where latching mode is not required. As expected, similar behavior was obtained for $|I_{\mathrm{SD}}|$ smaller than the retrapping current I_{R} ($I_{\mathrm{R}} = 1.1$ μA in the present device). Figure 6 demonstrates switching operation as a function of I_{SD}. For $|I_{\mathrm{SD}}| < 1$ μA clear and fast switching operation was obtained, without the need of self-resetting the device at every gate cycle. On the contrary, for $|I_{\mathrm{SD}}| > I_{\mathrm{R}}$ no switching was observed.

* email: afu@ibm.zurich.com
† email: fni@ibm.zurich.com

[1] Wagner, A., Ranzani, L., Ribeill, G. & Ohki, T. A. Demonstration of a superconducting nanowire microwave switch. *Appl. Phys. Lett.* **115**, 172602 (2020).

[2] McCaughan, A. N. & Berggren, K. K. A superconducting-nanowire three-terminal electrothermal device. *Nano Lett.* **14**, 5748–5753 (2014).
